# Repair of an Extensive Furcation Perforation with CEM Cement: A Case Study 

**Published:** 2013-12-24

**Authors:** Mohammad Jafar Eghbal, Mahta Fazlyab, Saeed Asgary

**Affiliations:** a * Dental Research Center, Research Institute of Dental sciences, Shahid Beheshti University of Medical Sciences, Tehran, Iran; *; b * Iranian Center for Endodontic Research, Research Institute of Dental sciences, Shahid Beheshti University of Medical Sciences, Tehran, Iran*

**Keywords:** Biocompatibility, Calcium Enriched Mixture, CEM Cement; Dental Cements, Endodontics, Furcation Perforation, Root Canal Therapy, Treatment Outcome

## Abstract

Iatrogenic perforation of the furcation area in multi-rooted molars during preparation of the access cavity can potentially lead to tooth extraction. The present case report describes the nonsurgical endodontic management of an extensive pulp chamber floor perforation in a first mandibular molar with calcium enriched mixture (CEM) cement. The perforation was chemically cleaned and then physically sealed with CEM cement. Root canal therapy was completed and the tooth was then restored with amalgam. A one-year follow-up revealed the absence of symptoms of infection/inflammation as well as clinical and radiographic signs/symptoms and therefore, can be interpreted as a favorable treatment outcome.

## Introduction

Iatrogenic perforation of pulp chamber floor is an undesirable complication in dental practice that can have a negative impact on the treatment prognosis [1, 2]. The size and level/location of the perforation as well as the time interval between the accident and its repair will influence prognosis [2].

The ideal (bio)material for perforation repair should be antibacterial [3], radiopaque, non-cytotoxic, non-absorbable, biocompatible and able to induce formation of hard tissue, particularly cementum, over the material [4] and also provide a three-dimensional seal [4-6]. A wide range of materials have been suggested for surgical and nonsurgical repair of perforations including zinc oxide eugenol, calcium hydroxide, Cavit, amalgam, glass ionomer, composite resin, mineral trioxide aggregate (MTA) [4, 7] and calcium enriched mixture (CEM) cement [4].

CEM cement is a water-based tooth-colored biomaterial with variable applications in endodontics [8]. Several studies indicate that CEM cement has antibacterial and antifungal effects [9], provides a physical and biological seal [10], is non-toxic and biocompatible [8, 11, 12], and able to induce osteogenesis [8], dentinogenesis [13] and cementogenesis [4, 8].

This article represents a case of an unusual extensive perforation of the pulp chamber floor in a first mandibular left molar and its subsequent repair with CEM cement.

## Case Report

**Figure 1:**
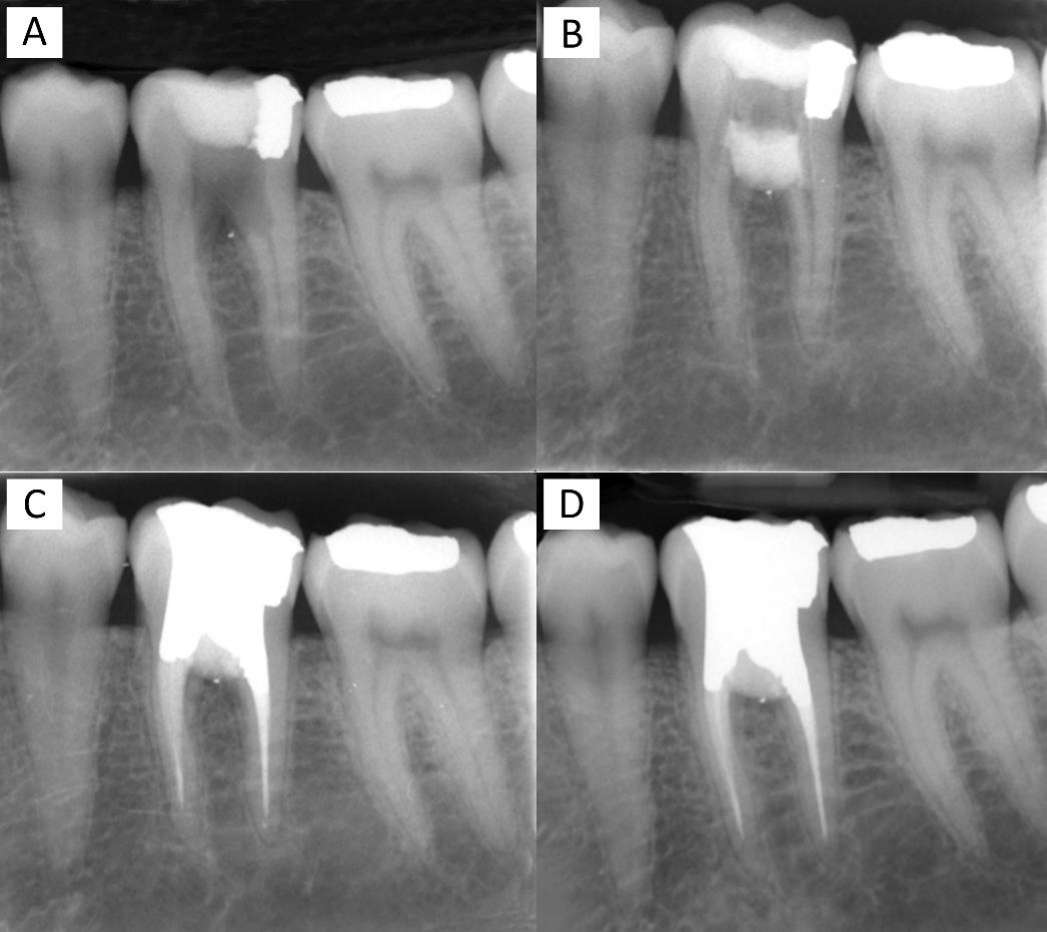
*A)* Preoperative periapical radiograph of a mandibular left first molar showing an unusual extensive furcation perforation, note the radiopaque small piece of amalgam indicating the apical extend of the defect into the bone; *B)* Postoperative periapical radiograph of the molar showing perforation repair; *C)* The molar tooth after root canal therapy and coronal amalgam restoration; *D)* One-year follow-up radiograph showing no radiolucency at the furcal area

A healthy Caucasian 28-year old female was referred to our dental clinic with a continuous dull pain in the left mandibular region, which had started after initiation of root canal treatment on the first molar by her general dentist about two weeks before. Upon clinical examination, a localized inflammation overlying the buccal mucosa in furcal region of the tooth #36 became evident which was tender on palpation. The tooth had no tenderness on percussion and no mobility. Careful periodontal probing showed a pocket depth within the normal range (<3 mm). Radiographic examination revealed overextended endodontic access cavity preparation and extensive destruction of pulp-chamber floor (Figure 1A).

In the radiography a small piece of amalgam was evident in the apical extend of the perforation which is believed to have separated from the previous amalgam restoration of the tooth. The location of this small piece was an indicator of wide destruction of the interradicular bone during the preparation of the access cavity (Figure 1A). 

The possible treatment options, including tooth extraction with/without replacement, tooth hemisection and non-surgical perforation repair with subsequent continuing of the root canal therapy (RCT) and coronal restoration were explained for the patient. The patient preferred the option of saving the tooth via a non-surgical endodontic treatment and furcal perforation repair with CEM cement. She also read and signed an informed consent.

Before the start of the treatment, mouth rinse with 0.2% chlorhexidine rinse (Behsa Co., Tehran, Iran) was carried out in order to control the oral microbial flora. After administering local anesthesia (2% lidocaine with 1:80000 adrenalin; Daroupakhsh, Tehran, Iran) and proper tooth isolation, the temporary restorative material was removed with a slight correction in the outlines of access cavity and then all canal orifices were located. The cavity and perforation site were copiously irrigated with full concentration of sodium hypochlorite and then normal saline. All canal orifices were preflared with #2 and 3 Gates-Glidden drills. Then the canals were blocked with appropriate-sized gutta-percha points to avoid obstruction with perforation repair material. CEM cement (BioniqueDent, Tehran, Iran) was prepared according to manufacturer's instruction and was placed into the cavity. In order to obtain a good marginal adaptation, the bulk of biomaterial was gently packed with a dry cotton pellet. After a few minutes required for initial setting of CEM, the gutta-percha points were removed and the biomaterial was covered with a moistened cotton pellet. After application of temporary restoration (Figure 1B), the patient was dismissed.

The second visit was a week later during which the patient was clinically evaluated. All the sign/symptoms had subsided and the tooth underwent conventional non-surgical root canal treatment. The working length was established and all four canals (naming distobuccal/lingual and mesiobuccal/lingual) were mechanically shaped using modified step-back technique and chemically cleaned by means of copious irrigation with 5.25% sodium hypochlorite and then normal saline. The root canals were filled with gutta-percha points (Ariadent, Tehran, Iran) and Roth root canal sealer (Roth's 801, Elite Grade; Roth Int., Chicago, IL, USA) using lateral condensation technique; the tooth was then restored with amalgam in the very same session. After taking the postoperative radiography (Figure 1C), the patient was put on a scheduled follow-up.

During the one year follow-up, the tooth remained functional and asymptomatic. Clinical examination showed that the tooth had no tenderness to percussion/palpation and the probing depth remained within normal level. Radiographic examination demonstrated adequate filling and sealing of the perforation site with normal periodontal apparatus (Figure 1D).

## Discussion

The present article reported the non-surgical repair of an extensive perforation in the pulp chamber floor of a mandibular molar with notable bone destruction, using CEM biomaterial. 

Many issues can affect the prognosis of furcation perforations, including the level and location of the lesion, size of the perforated area, as well as the time elapsed between the accident and its repair [14, 15]. While the location of furcal perforations (in case of surgical access) and the level of perforation in relation to the crestal bone are challenging, the size and shape of a perforation and the time of repair, are additional prognostic factors [16]. Extended defects are associated with lower prognosis and it is proved that the longer the time elapse before lesion repair, the poorer the prognosis, which is believed to be due to formation of the junctional epithelium to the most apical border of the perforation, a chronic inflammatory reaction characterized by formation of granulation tissue, that may lead to irreversible attachment and bone loss [14, 16]. In the present case, although pulp chamber floor perforation and interradicular bone destruction was very extensive and the time between perforation and repair was ~2 weeks, the treatment was clinically and radiographically successful and the tooth continued to stay functional during the one year recall.

A tightly sealed repair in the perforation site is the key to successful treatment as it disrupts path of microbial contamination and guards the periodontium apparatus for optimal healing [4, 15]. It is stated that mineral trioxide aggregate is not only able to create a biocompatible barrier against which the repair material can be packed, but also it acts as a repair material itself that provides a physical seal when applied in perforation repair [4, 17]. CEM cement proved to be similarly and reliably applicable, both as a barrier and repair biomaterial [4, 18, 19]; also CEM cement stimulates the formation of an additional biological seal via hydroxyapatite sediments at the interface between the biomaterial and soft vital tissue [4, 8, 10].

In cases of extensive furcal perforations, an internal matrix (barrier) technique has been suggested; albeit the defect should be directly accessible for the successful use of this technique. The matrix should be sterile and non-irritant and easily manipulated [20]. Hydroxyapatite, calcium sulfate, hydroxyapatite calcium sulfate, decalcified freeze-dried bone and combination of resorbable collagen and MTA are suggested for this technique [21]; materials in which calcium hydroxide is a main ingredient, are not suitable for crestal and furcation perforations due to subsequent pocket formation [22, 23]. We employed CEM cement as both the internal matrix and also the repair material, with favorable treatment outcome.

The desirable chemical and physical characteristics of CEM make it an optimal biomaterial for repairing the furcal perforations [4]. Studies regarding the *in vitro* and *in vivo* biocompatibility of CEM [24, 25], revealed not only its non cytotoxicity and non mutagenic potential [8, 11], but also its ability to induce the formation of mineralized tissue. CEM cement is proved to be a biologically active substrate for induction of hard tissue formation by osteoblasts (osteogenesis) [26] and cementoblasts (cementogenesis) [4]. The rather low levels of indigenous calcium, phosphate and hydroxyl ions leaching out from the biomaterial, is believed to be responsible for this bioactivity [8, 11].

In the presented case no radiographic and clinical sign/symptoms of disease in the bifurcation area was evident after a one-year follow-up; accordingly, CEM cement may be a suitable endodontic biomaterial for the non-surgical repair of extensive furcal perforations.

## Conclusion

The favorable treatment outcomes of this case confirms that CEM cement is a cementogenic and osteogenic biomaterial with good biocompatible sealing properties, and is ideal for use as a barrier and repair material in cases of teeth with extensive furcal perforation. 
